# A Metabolomic Analysis of Cirrhotic Ascites

**DOI:** 10.3390/molecules27123935

**Published:** 2022-06-20

**Authors:** Diren Beyoğlu, Cedric Simillion, Federico Storni, Andrea De Gottardi, Jeffrey R. Idle

**Affiliations:** 1 Arthur G. Zupko Institute for Systems Pharmacology and Pharmacogenomics, Arnold and Marie Schwartz College of Pharmacy and Health Sciences, Long Island University, Brooklyn, New York, NY 11201, USA; diren.beyoglu@liu.edu; 2 Hepatology, Department of BioMedical Research, University of Bern, 3008 Bern, Switzerland; 3 Hoffmann-La Roche AG, 4070 Basel, Switzerland; cedric.simillion@roche.com; 4 Department of Visceral Surgery and Medicine, Inselspital, University Hospital Bern, 3010 Bern, Switzerland; federico.storni@insel.ch; 5 Faculty of Biomedical Sciences, Università della Svizzera Italiana, 6900 Lugano, Switzerland; andrea.degottardi@usi.ch; 6 Gastroenterology and Hepatology, Ente Ospedaliero Cantonale, 6500 Lugano, Switzerland

**Keywords:** ascites, decompensated cirrhosis, metabolomics, amino acids, fatty acids, cholesterol

## Abstract

Ascites is a common complication of decompensated liver cirrhosis, and yet relatively little is known about its biochemical composition. We conducted two metabolomic investigations, comparing the profile of ascites from 33 cirrhotic patients and postoperative peritoneal drainage fluid from 33 surgical patients (Experiment 1). The profile of paired ascites and plasma was also compared in 17 cirrhotic patients (Experiment 2). Gas chromatography–mass spectrometry-based metabolomics identified 29 metabolites that significantly characterized ascites fluid, whether postoperative drainage fluid or plasma were used as controls. Ten elevated amino acids (glutamine, proline, histidine, tyrosine, glycine, valine, threonine, methionine, lysine, phenylalanine) and seven diminished lipids (laurate, myristate, palmitate, oleate, vaccenate, stearate, cholesterol) largely comprised the cirrhotic ascites metabolomic phenotype that differed significantly (adjusted *p* < 0.002 to 0.03) from peritoneal drainage fluid or plasma. The pattern of upregulated amino acids in cirrhotic ascites did not indicate albumin proteolysis by peritoneal bacteria. Bidirectional clustering showed that the more severe the cirrhosis, the lower the lipid concentration in ascitic fluid. The metabolomic compartment of ascites in patients with decompensated cirrhosis is characterized by increased amino acids and decreased lipids. These novel findings have potential relevance for diagnostic purposes.

## 1. Introduction

Liver disease is responsible for about two million deaths per year worldwide. Up to half of these deaths are due to complications of liver cirrhosis (LC), the 11th most common cause of death globally [1]. LC is the final stage of progressing fibrosis that is mainly due to excess alcohol consumption, obesity or viral hepatitis. Even with cirrhosis, the liver can function normally, and this state is known as compensated cirrhosis. However, an acute deterioration of liver function in a patient with compensated cirrhosis can lead to decompensated cirrhosis, when jaundice, ascites, hepatic encephalopathy, hepatorenal syndrome or variceal hemorrhage develop [2]. Approximately 60% of cirrhotic patients develop ascites within 10 years of diagnosis [3]. Ascites is usually treated with a low-sodium diet and administration of diuretics such as furosemide and spironolactone, and with paracentesis to physically remove the accumulated fluid in the peritoneum [3]. Attempts to distinguish cirrhotic, malignant, cardiac and inflammatory origins of ascites go back to the 1950s with paper chromatography used to measure proteins such as albumin, α1, α2 and ɣ-globulin fractions [4], followed by radial immunodiffusion introduced in 1965. A total of 23 plasma proteins in ascites were determined using this methodology [5]. Lipoproteins [6] and bile acids [7] in ascites have also been determined in previous studies. In the new era of mass spectrometry, a single study into the composition of cirrhotic ascites has been reported [8].

There are several reports on the background of metabolomics [9] and its use in biomarker definition [10,11] and the discovery or elucidation of biochemical networks and mechanisms [12]. A working definition of metabolomics would be that previously reported by [9,11]: “metabolomics studies the low molecular weight metabolites [e.g., <1.5 kDa] found in cells and organisms, usually through the analysis of plasma/serum, urine or cell culture medium using mainly MS or NMR technologies.” The different ways in which metabolomic investigations can be conducted have been discussed in detail by us and others [10,11]. Mass spectrometry provides a means of conducting metabolomics investigations and, as has been described [13], gas chromatography–mass spectrometry (GC-MS) offers a convenient platform for metabolomics investigations. This technology was employed to examine ovarian cancer ascites using cirrhotic ascites as a control. Fourteen metabolites varied between these two groups, with seven elevated in ovarian cancer ascites (mostly fatty acids) and seven elevated in cirrhotic ascites (mostly sugars) [14]. Ovarian cancer ascites was further investigated using human and mouse ovarian cancer cells, both in culture and xenotransplanted into mice. Differences in the metabolic composition of ascitic fluid between the mouse and human transplanted cells were reported using high-resolution ^1^H NMR [15]. This same technique was employed to investigate ascites associated with both reactive ovarian carcinoma and hepatocellular carcinoma (HCC). The ovarian carcinoma ascites was enriched in alanine, phenylalanine and lipids and depleted in glutamine [16]. A study of serum from cirrhotic ascites patients and healthy controls was reported, in which 12 amino acids and 8 conjugated bile acids were significantly increased in the ascites patients [17].

The limited and heterogeneous reports involving cirrhotic ascites teach us little about the biochemical composition of ascites and in relation to clinical variables. Nor do these studies identify potential mechanisms for how small organic molecules enter ascitic fluid. We therefore conducted a metabolomic investigation of ascites fluid from 33 cirrhotic patients and of peritoneal drainage fluid from 33 postoperative patients as controls (Experiment 1). Additionally, we made a metabolomic comparison between paired plasma and ascites fluid in 17 of these cirrhotic patients (Experiment 2). The study is both of clinical and scientific significance because it demonstrates, in two separate investigational arms, how the peritoneal fluid compartment is metabolically altered in cirrhotic ascites. The elevation of amino acids and diminution of fatty acids in ascites may reveal new insights into the formation of ascites and have potential relevance for diagnostic purposes.

## 2. Results

### 2.1. Experiment 1. Cirrhotic Ascites vs. Postoperative Peritoneal Drainage Fluid

**Figure 1:**
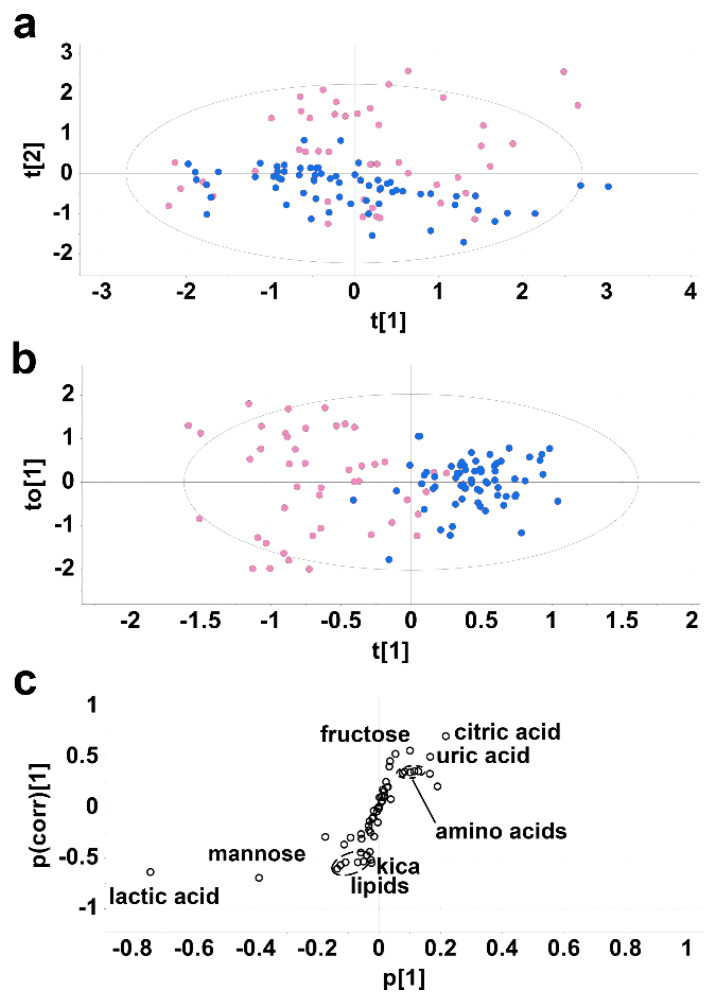
Multivariate data analysis of ascites metabolites vs. postoperative surgical drainage fluid metabolites. (**a**) Unsupervised PCA scores plot showing partial separation of cirrhotic ascites samples (blue) and surgical postoperative drainage samples (pink). Each point represents a sample analyzed by GC-MS. The Hotelling’s ellipse represents the 95% confidence boundary. (**b**) Supervised OPLS-DA scores plot showing clustering and separation of ascites and surgical drainage samples. (**c**), OPLS-DA loadings S-plot showing metabolites upregulated in ascites fluid (top right) and metabolites downregulated in ascites relative to control drainage fluid (bottom left). The *X*-axis (p[1]) represents the variable magnitude and the *Y*-axis (p(corr)[1]) represents the reliability of the variable.

Analysis by gas chromatography–mass spectrometry (GC-MS) of cirrhotic ascites fluid versus postoperative peritoneal fluid confirmed the presence of 58 metabolites, identified according to matching mass spectra with a spectral database and in direct comparison with an in-house library of authentic standards. Multivariate data analysis revealed a partial separation with little clustering for the 33 ascites patients vs. the 33 control postoperative peritoneal drainage patients in the principal components analysis (PCA) scores plot (Figure 1a). The top three components of variance were chosen (PCA1-3) and scores plots were created for PCA1 vs. PCA2, PCA1 vs. PCA3 and PCA2 vs. PCA3, each of which shows the 95% confidence ellipse around each group (Figure S1). The sum of squares explained by the PCA model (R2) was 0.609 and the predicted residual sum of squares (Q2) was 0.070. A supervised projection to a latent structures-discriminant analysis (PLS-DA) model was generated and found to be valid using a leave-one-out cross validation, whereby R2 and Q2 decreased to <0.3 and <0, respectively (Figure S2). In addition, an ANOVA cross validation was performed and found to be significant with F = 35.8 and P = 1.85 × 10^−19^. Using an orthogonal projection to a latent structures-discriminant analysis (OPLS-DA) model, the separation between ascites fluid samples and controls was much improved compared to the PCA (Figure 1b). The OPLS-DA loadings S-plot (Figure 1c) revealed metabolites that were both augmented and diminished in cirrhosis fluid relative to control postoperative drainage fluid. Of these, 24 differed significantly (adjusted *p* values < 0.002 to 0.007) between ascites and control peritoneal drainage fluid. These metabolites are listed in Table 1. Of these 24 metabolites, 11 had not previously been described in cirrhotic ascites fluid.

Of particular note were elevated amino acids and lowered lipids in ascites. Further details of these metabolites are given in Table S1. Citric, glycolic and uric acids were all elevated in ascites together with the amino acids, glutamine, proline, histidine and tyrosine and the lysine metabolite pipecolic acid. The amino acids were elevated in a tight cluster (Figure 1c), suggesting that they all appeared in ascites via a related mechanism. Among sugars and their metabolites, erythritol, *myo*-inositol, *scyllo*-inositol and fructose were all elevated in ascites. Diminished in ascites were the lipids lauric, myristic, palmitic, linoleic, oleic and stearic acids, together with cholesterol. These lipids also appeared as a tight cluster in the OPLS-DA loadings S-plot (Figure 1c), again suggesting their downregulated concentrations in ascites were due to a common mechanism. In addition, the sugars mannose and gluconic acid were both reduced in ascites, together with glutamic acid and the leucine metabolite 2-ketoisocaproic acid (kica). Finally, there was a lower level of lactic acid in ascites than in control drainage fluid. When the etiology of the underlying cirrhosis was analyzed as two groups, metabolic (alcoholic and non-alcoholic steatohepatitis) vs. viral and autoimmune hepatitis, it was found that erythritol was significantly higher in the metabolic group than the viral/autoimmune hepatitis group (*p* < 0.01). No other metabolite differed between these two etiological groups.

Two general features of this analysis are of note. Firstly, the separation of ascites and surgical drainage samples in the PCA and OPLS-DA scores plots (Figure 1a,b) is incomplete. Nevertheless, this separation was significant based upon the validation of the model [18,19,20], where R2 decayed to <0.3 and Q2 to <0 (Figure S3A). Secondly, the degree of variance within the control drainage samples is greater than that for the ascites samples (Figure S1B), reflecting the heterogeneous nature of the postsurgical drainage patients and modes of peritoneal drainage employed. These findings comparing cirrhotic ascites with peritoneal drainage simply show the broad range of metabolites that are significantly elevated or attenuated (adjusted *p* values < 0.002 to 0.007) in ascites compared to peritoneal drainage. The generalized pattern that emerged from Experiment 1 was one of elevated amino acids and diminished lipids in cirrhotic ascites fluid. This was further validated using ROC analysis (Figure S4A), whereby the combination of elevated amino acids in cirrhotic ascites fluid glutamine, proline, histidine and tyrosine (Table 1) yielded a significant (*p* < 0.0001) ROC curve with an AUC of 0.74. Moreover, the combination of diminished lipid species lauric, myristic, palmitic, linoleic, oleic and stearic acids, together with cholesterol, also yielded a significant (*p* < 0.0001) ROC curve with an AUC of 0.90.

### 2.2. Experiment 2. Cirrhotic Ascites vs. Paired Plasmas

**Figure 2:**
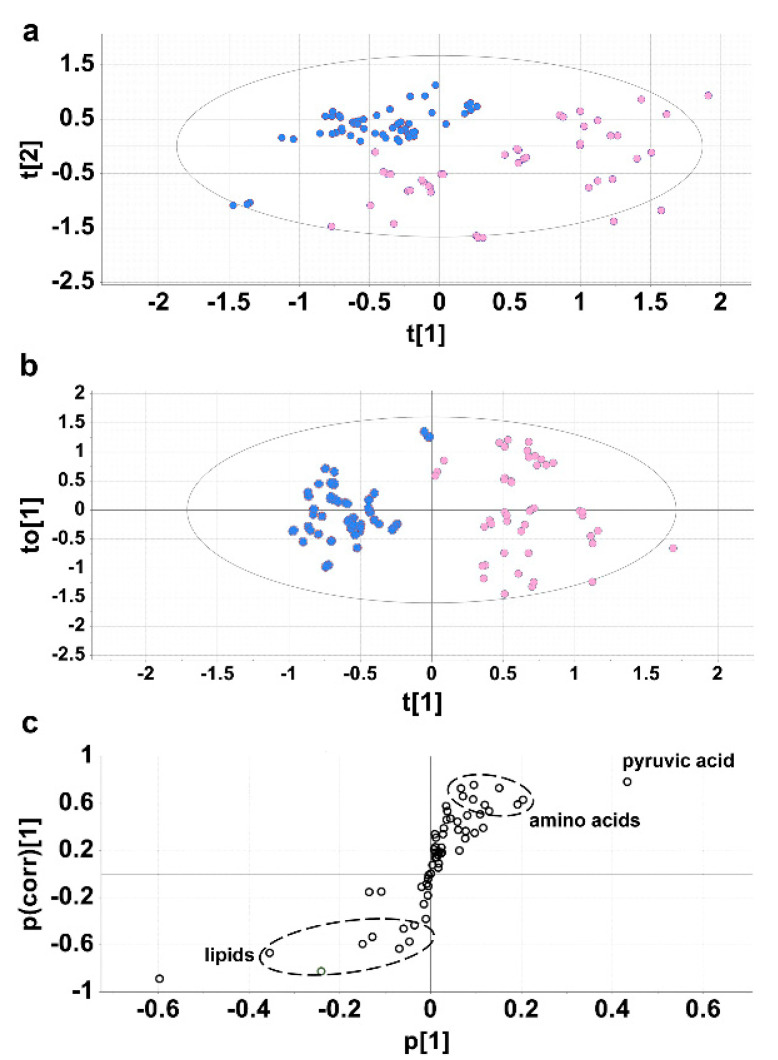
Multivariate data analysis of ascites metabolites vs. paired plasma metabolites. (**a**) Unsupervised PCA scores plot showing partial separation of cirrhotic ascites fluid (blue) and plasma samples (pink) from the same patients. Each point represents a sample analyzed by GC-MS. (**b**) Supervised OPLS-DA scores plot showing clustering and separation of cirrhotic ascites fluid and plasma samples. (**c**) OPLS-DA loadings S-plot showing metabolites upregulated in ascites fluid (top right) and metabolites downregulated in ascites samples relative to paired plasma samples (bottom left).

For 17 of the ascites patients, the plasma metabolome was also investigated using GC-MS and compared with the ascites fluid metabolome. Figure 2a shows a PCA scores plot in which the ascites samples (blue) and the plasma samples (pink) completely resolved, although clustering of each group was weak. The top three components of variance were chosen (PCA1-3) and scores plots for PCA1 vs. PCA2, PCA1 vs. PCA3 and PCA2 vs. PCA3, each of which shows the 95% confidence ellipse around each group (Figure S3). The sum of squares explained by the PCA model (R2) was 0.617 and the predicted residual sum of squares (Q2) was 0.082. A supervised projection to a latent structures-discriminant analysis (PLS-DA) model was generated and found to be valid using a leave-one-out cross validation, whereby R2 and Q2 decreased to <0.3 and <0, respectively (Figure S2B). In addition, an ANOVA cross validation was performed and found to be significant with F = 104 and P = 3.82 × 10^−33^. In the OPLS-DA model, the scores plot (Figure 2b) showed clear separation between ascites and plasma samples with reasonable clustering. The OPLS-DA loadings S-plot (Figure 2c) shows pyruvic acid plus a group of nine amino acids that clustered together and were upregulated in ascites fluid relative to plasma and a number of lipid molecules that also clustered together and were downregulated in ascites fluid relative to plasma. Details of the 19 metabolites that differed between ascites fluid and plasma drawn from the same 17 cirrhotic patients are given in Table 2. Seven of these metabolites had not previously been reported in cirrhotic ascites. A total of 29 metabolites differed significantly (adjusted *p* values < 0.002 to 0.007) between cirrhotic ascites and controls (postoperative drainage fluid or cirrhotic ascites patient plasma) and details of these are given in Table S1. Of the nine elevated amino acids in ascites relative to plasma, three (glutamine, proline and tyrosine) were the same as those elevated in ascites relative to postoperative peritoneal drainage fluid (Table 1). None of these nine were true non-essential amino acids; rather, they were either essential (valine, lysine, methionine, threonine and phenylalanine) or conditionally essential (tyrosine, glutamine, proline and glycine) [21]. The downregulated lipids comprised three saturated fatty acids, two monounsaturated fatty acids plus cholesterol (Table 2). Of these six lipid molecules, five were identical to the downregulated lipid species in ascites fluid relative to postoperative peritoneal drainage fluid (Table 1). In total, 16 novel metabolic constituents of cirrhotic ascites are described herein. Note that the elevated amino acids and the decreased lipids were clustered, again suggesting common mechanisms for their entry into ascites.

Accordingly, Experiment 1 and Experiment 2 produced remarkably similar findings despite the vastly different study designs that used diverse controls. The metabolic phenotype of cirrhotic ascites has therefore been defined from two differential experimental perspectives.

### 2.3. Bidirectional Clustering of Metabolomic Data Versus Clinical Data

**Figure 3:**
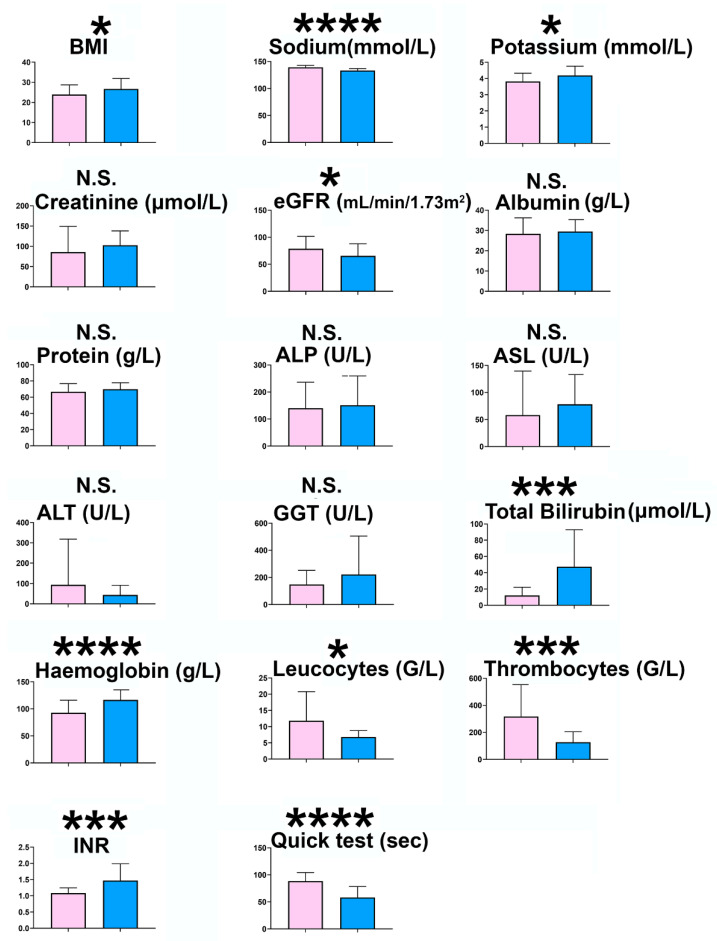
Clinical variables for 33 cirrhotic ascites patients (blue) and 33 surgical postoperative drainage patients (pink) shown as means ± SD. Statistical significances are represented by *, *p* < 0.05; ***, *p* < 0.001; ****, *p* < 0.0001.

**Figure 4:**
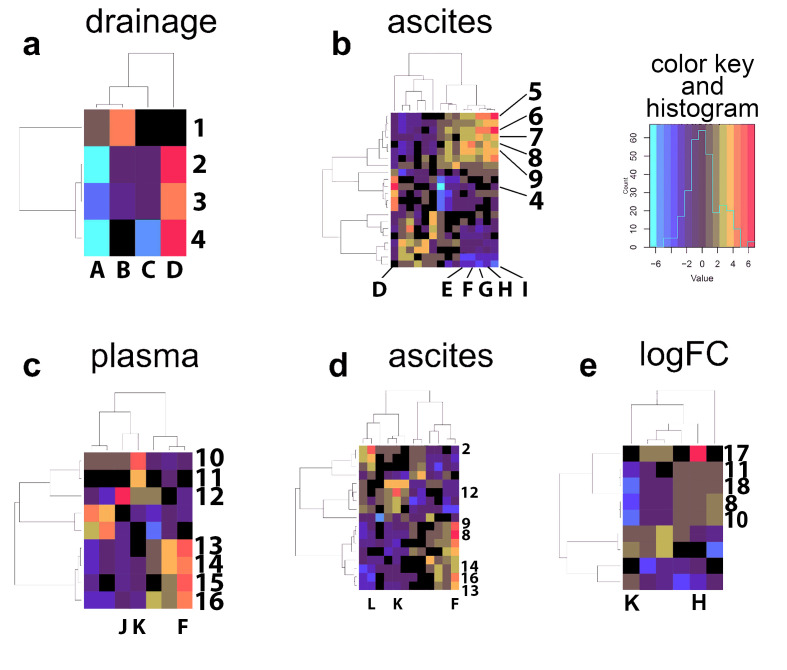
Cluster maps showing bidirectional clustering of clinical covariates from Experiment 1; (**a**), metabolites in drainage fluid for postoperative surgical patients (n = 33); (**b**), metabolites in cirrhotic ascites fluid (n = 33); from Experiment 2: (**c**), metabolites in paired plasma (n = 17); (**d**), metabolites in paired cirrhotic ascites fluid (n = 17); (**e**), metabolites as log fold concentration (logFC) difference between cirrhotic ascites fluid and paired plasma (n = 17) in Experiment 2. **Abbreviations:** 1—galactose; 2—erythritol; 3—*myo*-inositol; 4—2,3-dihydroxybutanoic acid; 5—cysteine; 6—2-ketoisocaproic acid; 7—cholesterol; 8—palmitic acid; 9—stearic acid; 10—vaccenic acid ((11*E*)-11-octadecenoic acid); 11—myristic acid; 12—2-hydroxy-3-methylbutanoic acid; 13—leucine; 14—valine; 15—2-aminobutanoic acid; 16—isoleucine; 17—tyrosine; 18—oleic acid; A—estimated glomerular filtration rate; B—Quick’s test (prothrombin time); C—sex; D—plasma creatinine concentration; E—body weight; F—body mass index; G—thrombocytes; H—plasma albumin concentration; I—total plasma protein; J—aspartate aminotransferase; K—Child–Pugh score; L—age. A key to the colors and their distribution is also given.

Bidirectional clustering of all data was conducted by displaying demographic and clinical variables (see Figure 3) and metabolite concentrations on the *X* and *Y* axes, respectively. Two cluster maps were derived from data generated in Experiment 1 (Figure 4a,b). Analysis of the metabolic profile versus clinical variables for the postoperative peritoneal drainage fluid produced a simple cluster map with few correlations (Figure 4a). Three metabolites, erythritol, *myo*-inositol and 2,3-dihydroxybutanoic acid, in drainage fluid correlated positively (red/orange) with plasma creatinine concentration (D) and therefore, not unexpectedly, negatively (cyan/blue) with the estimated glomerular filtration rate (A). The cluster map for ascites fluid from Experiment 1 contained considerably more associations between clinical variables and metabolite levels (Figure 4b). Of note is a cluster containing three lipids (cholesterol (7), palmitic acid (8) and stearic acid (9)), cysteine (5) and the leucine metabolite 2-ketoisocaproic acid (6). This cluster is positively correlated with plasma protein (I), plasma albumin (H), thrombocytes (G), body mass index (F) and body weight (E). Two cluster maps also originated from the data of Experiment 2 (Figure 4c,d).

The cluster map for plasma (Figure 4c) contained an area in which body mass index (F) was positively correlated with all three branched-chain amino acids, valine (14), leucine (13) and isoleucine (16), together with 2-aminobutanoic acid (15), a potential intermediate in isoleucine synthesis from threonine [22]. Additionally, two fatty acids, vaccenic (10) and myristic acids (11) were positively correlated with the Child–Pugh score (K). Interestingly, 2-hydroxy-3-methylbutanoic acid (2-hydroxyisovaleric acid; 12) was strongly positively correlated with plasma aspartate transaminase (J) concentrations (Figure 4c). It has been reported that 2-hydroxy-3-methylbutanoic acid is derived from degradation of branched-chain amino acids (BCAAs) [23] but that BCAAs are not metabolized by aspartate transaminase [24]. The cluster map for ascites fluid from Experiment 2 had a similar but stronger correlation of body mass index (F) with stearic (9) and palmitic acids (8) and a positive correlation with all three BCAAs (13, 14, 16; Figure 4d). Unlike plasma, 2-hydroxy-3-methylbutanoic acid (12) was correlated with the Child–Pugh score (K) for ascites fluid. The erythritol (2) level in ascites fluid was strongly correlated with patient age (L). For Experiment 2, the log fold concentration difference between ascites and plasma (logFC) was expressed in a cluster map with clinical covariates (Figure 4e). Two main features were observed. Firstly, a strong correlation between plasma albumin concentration (H) and the logFC of tyrosine (17) and secondly, a strong negative correlation between Child–Pugh score and the four lipids myristic (11), oleic (18), palmitic (8) and vaccenic acids (10), most prominently with the saturated fatty acids myristic (11) and palmitic acids (8). A heatmap for all 17 ascites samples and all 17 plasma samples from Experiment 2 that includes all metabolite features detected is given in Figure S5.

## 3. Discussion

Two investigations have been conducted with the aim of defining the metabolomic phenotype of cirrhotic ascites fluid. In Experiment 1, where cirrhotic ascites fluid was compared with surgical postoperative drainage fluid, it was established that the concentration of 24 metabolites (11 not previously described in cirrhotic ascites fluid) was significantly different (adjusted *p* values < 0.002 to 0.007) between these two groups. The fundamental metabolic phenotype of cirrhotic ascites was elevated amino acids and diminished lipids. In Experiment 2, where cirrhotic ascites fluid was compared with plasma from the same patients, the concentration of 19 metabolites (7 not previously described in cirrhotic ascites fluid) was significantly different (adjusted *p* values 0.002 to 0.03) between paired ascites fluid and plasma. Interestingly, the metabolic phenotype of cirrhotic ascites in both investigations was elevated amino acids and diminished lipids.

Regarding the diminution of fatty acids and cholesterol in cirrhotic ascites relative to either postoperative drainage fluid or plasma, it is proposed that these observations can in part be interpreted in terms of the concentration and physicochemical properties of albumin. Cirrhotic ascites fluid is a transudative ascites due to portal hypertension with a serum–ascites albumin gradient (SAAG) ≥ 11 g/L [25]. The mean serum albumin concentration of the cirrhotic patients in this study was 27.4 g/L and therefore the ascites albumin concentration would be expected to be ≤16.4 g/L; that is, ~60% of the serum albumin concentration. This diminished concentration of ascitic albumin will result in a reduced concentration of fatty acids due to the strong correlation between the two [26] and the strong binding of fatty acids to albumin [27] that occurs at up to seven binding sites per albumin molecule [28]. It is clear that during the sample workup in our GC-MS investigations, the fatty acids that are bound by nonpolar interactions between the fatty acid hydrocarbon chains and the uncharged amino acid side chains that comprise the albumin binding grooves [29,30,31,32] will be stripped off. Multiple authors have investigated the binding affinities of various fatty acids to albumin, and their findings support the conclusion that diminished concentrations of saturated, monounsaturated and diunsaturated fatty acids in ascites fluid result from the low concentration of albumin in transudative ascites fluid. Moreover, the observed ratios in both experiments of ascites/control fatty acid concentrations of 0.1–0.4 are generally consistent with the decrease in albumin concentration in ascites.

Regarding the increased concentration of α-amino acids in ascites relative to both postoperative peritoneal drainage fluid and plasma, the observations cannot be attributed directly to the difference in albumin concentrations. Plasma concentrations of amino acids are not only subject to homeostatic control, but are also affected by many variables, including habitual diet [33], metabolism, lifestyle and genetic factors [34]. There have been several previous reports of amino acid plasma concentrations in decompensated cirrhosis with ascites, which in general showed that aromatic amino acids (phenylalanine, tyrosine and tryptophan) were elevated, while BCAAs were decreased in plasma [11]. These observations were consistent with the increased release of aromatic amino acids from peripheral tissues and an enhanced uptake from skeletal muscle of BCAAs for the purpose of ammonia detoxication (via synthesis of glutamine) in decompensated cirrhosis [35]. Therefore, the pattern of amino acids in serum is altered in decompensated cirrhosis. In the investigations described here, a total of ten amino acids were elevated in ascites fluid compared to either peritoneal drainage fluid or paired plasmas. One possibility was that peritoneal bacteria, found in 14/17 ascites fluids tested, caused the proteolysis of ascitic proteins, including albumin. However, the pattern of amino acids elevated in ascites fluid in the investigations described herein do not match the amino acid composition of albumin itself. For example, the most common of the 585 amino acid residues of human serum albumin comprise glutamate, aspartate, alanine and leucine [36], none of which were elevated in ascites fluid. Another possible explanation for elevated amino acids in ascites fluid is that in decompensated cirrhosis, the transcapillary escape rate of blood solutes is increased [37]. It is possible that plasma amino acids simply escape into ascites through mesenteric capillaries or across the surface of the liver. In such a case, the concentrations found in ascites should generally reflect those found in plasma. As stated above, aromatic amino acids are significantly elevated in plasma in patients with decompensated cirrhosis and ascites with a concomitant decline in BCAAs. While phenylalanine and tyrosine were elevated in ascites, tryptophan was not detected. Similarly, the BCAA valine was elevated in ascites. A study of the amino acid concentrations in ascites fluid and plasma of two dogs with liver cirrhosis was reported, in which only histidine and phenylalanine, of the ten amino acids reported here, displayed an increase in ascites over plasma [38]. It is deemed unlikely that elevated amino acids in ascites are related to their corresponding plasma concentrations. It is currently unknown if elevated amino acids in ascites fluid are somehow related to amino acid binding to albumin because it has been suggested that only L-tryptophan [39,40] and cysteine [39] bind to human serum albumin. Conversely, it was reported by several workers [41,42,43] that albumin tightly binds aromatic amino acids and transports them in the blood. One probable explanation for the increased concentration of amino acids and other small polar molecules in cirrhotic ascites fluid may be related to the mesenteric lymphatic system. The lymphatic vascular system normally reabsorbs excess fluid in the liver and splanchnic region. It is believed that the overwhelming of lymphatic drainage is a direct cause of ascites formation [44]. Under these conditions, it is highly probable that small molecules in lymph will easily pass into the ascites fluid, since lymphatic capillaries are highly permeable, being composed of a single layer of endothelial cells without a continuous basement membrane [44]. The composition of mesenteric lymph has been evaluated, together with plasma, using metabolomics. Approximately 150 out of the 269 metabolites characterized correlated (r > 0.7) between lymph and plasma. Among the strongest correlated metabolites were threonine (r = 0.997) and methionine (r = 0.984) [45]. The mechanism by which certain amino acids (Gln, Gly, His, Lys, Met, Phe, Pro, Thr, Tyr, Val) were significantly elevated in cirrhotic ascites fluid (area under ROC curve 0.89; *p* < 0.0001) is currently unknown but is unlikely to be due to bacterial degradation of albumin or the transport into ascites bound to albumin. Notably, the two amino acids most strongly correlated between plasma and mesenteric lymph, threonine and methionine [45] were among the amino acids most elevated in ascites fluid relative to plasma. This suggests that plasma amino acids may gain access to the ascites compartment via the mesenteric lymphatic system. Whatever the mechanism, it is clear that it applies to all the amino acids so far detected in cirrhotic ascites fluid (see above) because in both OPLS-DA loadings S-plots (Figure 1c and Figure 2c), the amino acids were tightly clustered.

The use of bidirectional clustering of demographic/clinical variables and metabolite concentrations provided novel insights into cirrhotic ascites. Firstly, the hotspots for serum creatinine concentration (crtn) with 2,3-dihydroxybutanoic acid (2,3-D) in both postoperative drainage fluid and ascites fluid may be related to their respective renal handling. This latter compound is the (2*R*,3*S*)-dihydroxybutanoic acid diastereomer, a metabolite of threonine and a putative oncometabolite in acute myeloid leukemia [13,46]. Its water solubility has been estimated as 454 g/L using artificial neural networks [47], considerably greater than creatinine water solubility (90 g/L). However, the structural isomer of 2,3-D, (*S*)-3,4-dihydroxybutanoic acid (2-deoxytetronic acid), an endogenous metabolite, dietary constituent and a metabolite of xylose produced by *E.coli* [48], has an estimated aqueous solubility of 835 g/L [47]. It has been reported for this isomer that the renal clearance roughly equals that of creatinine [49]. It is reasonable to propose therefore that 2,3-D and creatinine are handled similarly by the kidney, explaining why they are correlated.

Secondly, the strong correlation (hotspot) between plasma albumin and tyrosine concentrations in the logFC cluster map is of interest. A ^1^H NMR spectroscopy investigation of ascitic fluid has been reported, which showed differential metabolite patterns between malignant ascites (n = 15) and cirrhotic ascites (n = 47), groups that had SAAG values of 12 and 18 g/L (*p* < 0.01), respectively [50]. This indicates higher serum albumin in the cirrhotic ascites patients, who also had a significantly higher (*p* < 0.001) mean serum tyrosine concentration (124 ± 73 μM) than the malignant ascites patients (44 ± 17 μM). These findings are consistent with the cluster map observation that serum albumin and tyrosine concentrations are correlated.

Thirdly, another logFC feature of significance was the negative correlation (cold spot) between Child–Pugh score and myristic (14:0), oleic (18:1 9*Z*), palmitic (16:0) and vaccenic (18:1 11*E*) acids. These data can be interpreted as meaning that the more severe the cirrhosis, the lower the concentration of these fatty acids in ascitic fluid relative to plasma.

Animal models of ascites have furnished new knowledge regarding the mechanisms of ascites development. Intraperitoneal injection of carbon tetrachloride to rats induces decompensated liver cirrhosis with ascites [8]. The appearance of a modified serum amino acid profile in cirrhotic rats was largely prevented by application of an abdominal poultice containing the herbal compound-based formula *Xiaozhang Tie* (XT) [8,51]. XT application was reported to alter amino acid metabolism, particularly inducible NO synthase and neuronal NO synthase in the small intestine of cirrhotic rats. Pathway analysis demonstrated not only an effect of XT on Arg/Pro metabolism, but also on Tyr and Phe biotransformations [8]. A general case has been made for the metabolomic investigation of natural products in the treatment of liver disease in order to realize new mechanistic insights to assure the safety and efficacy of such products [51].

Better understanding of the pathophysiology of ascites formation and the metabolic parameters associated with it through metabolomic investigations may lead to the identification of novel drug targets for its treatment and/or prevention. Future work will need to address how the presence of diseases other than cirrhosis—for example, hepatocellular carcinoma or cholangiocarcinoma—influences the molecular composition of ascites fluid. The metabolite profile of ascites might therefore be used as an aid to determine the presence of malignancies within the liver.

## 4. Materials and Methods

### 4.1. Study Population

All patients gave their written informed consent to the study, which was conducted according to the World Medical Association Declaration of Helsinki and complied with the provisions of the Good Clinical Practice guidelines and local laws. The study was approved by the ethics committee of Canton Bern (Die Ethikkommission des Kantons Bern; reference 2107-02271). Exclusion criteria were age >80 or <18, insufficient biological samples or clinical and laboratory data and no informed consent. In Experiment 1, peritoneal fluid was collected either via abdominal paracentesis in patients with decompensated cirrhosis (n = 33) and from patients recovering from abdominal surgical interventions through peritoneal drain tubes (n = 33). In Experiment 2, plasma (4 mL; Li heparin) was prepared from 17 of the patients with decompensated cirrhosis around the time of paracentesis. Samples were aliquoted, a sample sent for analysis and the remainder stored at −20 °C until analyzed. The etiology of cirrhosis (no. of cases) was HCV (2), alcohol (13), HCV plus alcohol (2), alcohol plus Laennec’s (3), HBV (2), HBV plus alcohol (1), NASH (3), HBV plus NASH (1), HBV plus HCV plus alcohol (1), autoimmune (1), hemochromatosis (1), HBV plus autoimmune (1), primary biliary cholangitis (1) and Budd Chiari syndrome (1). The ascites and postoperative patients did not differ by age (59.0 ± 10.1; 60.2 ± 13.7 years, respectively) or by gender (12 F/22 M; 8 F/25 M, respectively). The cirrhotic patients were Child–Pugh (CP) class A (CP scores 6 and not recorded; n = 2), B (CP scores 7–9; n = 21) and C (CP scores 10–15; n = 10) and the postoperative drainage patients had passive drainage (n = 18), Easy Flow drain (n = 10), Pigtail drain (n = 1) and Jackson Pratt drain (n = 1), with one drain not recorded. Several postoperative drainage patients had carcinoma, including colorectal carcinoma (n = 6), pancreatic carcinoma (n = 3), gastric carcinoma (n = 1), metastatic melanoma (n = 1) and hepatobiliary carcinomas (n = 3). The body mass indices, serum biochemistry and hematology and liver function tests of the two patient groups are displayed and compared (mean ± standard deviation) in Figure 3. The cirrhotic ascites patients had significantly lower body mass index (*p* < 0.05), serum potassium (*p* < 0.05), estimated glomerular filtration rate (*p* < 0.05), leukocytes (*p* < 0.05), thrombocytes (*p* < 0.001) and Quick prothrombin test (*p* < 0.0001) and significantly higher serum sodium (*p* < 0.0001), total bilirubin (*p* < 0.001), hemoglobin (*p* < 0.0001) and INR (*p* < 0.001). Differences in liver enzymes ALT, AST, ALP and GGT were all non-significant, as were serum creatinine, glucose, total protein and albumin.

### 4.2. Gas Chromatography–Mass Spectrometry (GC-MS) Analysis

Aliquots of samples (50 µL) were subjected to GC-MS analysis with 4-chlorophenylacetic acid (2 mM; 100 µL) as internal standard using a slight modification of our published methods [18,19,20]. Samples were treated with an equal volume of ultrapure pyridine, then dried under nitrogen and derivatized with methoxyamine hydrochloride (MOX) and BSTFA/TMCS as described [18,19,20]. QC samples, prepared from aliquots of each sample, were included and analyzed as the first five samples injected and then interspersed between every tenth analytical samples. Samples (1.0 µL) were injected in duplicate using an Agilent 7683B liquid sampler into an Agilent 6890N gas chromatograph with an Agilent 5975B mass selective detector operating under electron impact ionization at 70 eV. The front inlet was operated in splitless mode at 250 °C and an HP5-MS column (60 m; i.d. 250 µm; film thickness 0.25 µm) subjected to a temperature program of 70 °C for 3 min, 10 deg/min to 250 °C, 10 deg/min to 300 °C, held for 8 min (run time 34 min). Mass spectra were collected from m/z 35.0 to 650.0. In each 34 min chromatogram, in addition to background peaks and the internal standard, 77 peaks deriving from 70 discrete metabolites (Table S1) were identified through comparison of their mass spectra with the NIST 14 Library (MS Wil GmbH, Wil, Switzerland), which contained 276, 248 mass spectra from 242, 466 compounds, and through comparison of their retention times with an in-house collection of 120 authentic standards. Each annotated peak was quantitated as a peak area ratio (PAR) from its peak area/internal standard peak area using AutoQuant in the on-board Agilent ChemStation software, followed by Quant Browser GCMS software (Leoson BV, Middelburg, The Netherlands). The resulting spreadsheet of PARs for each annotated metabolite was imported into SIMCA 17.0.0 (Sartorius Stedim Data Analytics AB, Umeå, Sweden). Multivariate data analysis using unsupervised PCA, supervised PLS-DA, with ANOVA cross validation and leave-one-out cross validation of the PLS-DA model, and OPLS-DA were conducted as described [18,19,20].

### 4.3. Statistics and Data Presentation

Multivariate data analysis conducted using SIMCA. Univariate statistics were performed using GraphPad Prism 9.3.1 (GraphPad Software, Inc., La Jolla, CA, USA). Means are expressed ± S.D. and *p* values are all two sided. For the demographic data and clinical measurements, an unpaired t test was used. For the metabolomic data, group differences were analyzed nonparametrically using Mann–Whitney U test and then subjected to Bonferroni correction to eliminate false discovery due to multiple comparisons [52]. For pairwise analysis of the metabolomic data between plasma and ascites, the Wilcoxon matched pair test was used. All statistical significances were expressed as two-tailed *p* values and adjusted for multiple comparisons using the Bonferroni correction. Receiver operating characteristic (ROC) curves were generated using GraphPad Prism 9.3.1.

Clustering analysis was performed as follows: each metabolite was tested for statistical association with each of the clinical covariates. For discrete or binary covariates, a two-sided Student’s t-test was used. For numerical covariates, a t statistic was calculated from the Pearson correlation coefficient. The values used for the metabolites were either the log-transformed intensities or the log-fold change (logFC) values between ascites and blood plasma. Next, a matrix was constructed with all the obtained t values for each pair of metabolites (rows) and covariates (columns). Rows and columns where all t values were less than 3.5 were considered non-informative and discarded. The rows and columns of the resulting matrix were clustered using hierarchical clustering using correlation distance as a distance metric. The results were visualized on a cluster map.

## 5. Conclusions

The investigation of cirrhotic ascites using GC-MS-based metabolomics identified 29 metabolites that significantly characterized ascites fluid compared with postoperative drainage fluid from surgical patients or from the cirrhotic patients’ own plasma. The metabolomic phenotype of cirrhotic ascites fluid principally comprised elevated amino acids and diminished fatty acids and cholesterol. The clustering of the amino acids, and also of the fatty acids, in the multivariate data analysis is suggestive of a single mechanism that facilitates entry of amino acids into ascites and a second common mechanism that constrains the entry of lipids into ascites. These novel findings have potential applicability to diagnostic determinations.

## Figures and Tables

**Table 1:** Metabolites showing a difference in relative concentration between ascitic fluid from cirrhotic patients and peritoneal fluid from postoperative surgical patients.

Metabolite (Reported Previously in Ascites; Y = Yes, N = No)	Control Peritoneal Fluid	Cirrhotic Ascites	* *p* Value	Ascites/ Control Ratio
METABOLIC INTERMEDIATES				
Lactic acid (Y)	1071	451	<0.002	0.4
Glycolic acid (N)	4	6	0.007	1.5
Citric acid (Y)	47	124	<0.002	2.6
Uric acid (Y)	62	138	<0.002	2.2
AMINO ACIDS AND AMINO ACID METABOLITES				
Glutamic acid (Y)	9	2	<0.002	0.2
Glutamine (Y)	36	61	<0.002	1.7
Proline (N)	113	149	0.005	1.3
Histidine (Y)	7	13	0.007	1.9
Tyrosine (Y)	58	107	<0.002	1.8
2-Ketoisocaproic acid (N)	2	0.8	<0.002	0.4
Pipecolic acid (N)	1	3	<0.002	3.0
SUGARS AND SUGAR METABOLITES				
Erythritol (N)	2	5	<0.002	2.5
Mannitol (N)	110	21	0.002	0.2
Gluconic acid (N)	3	2	<0.002	0.7
*Myo*-inositol (N)	20	42	<0.002	2.1
*Scyllo*-inositol (N)	1	5	<0.002	5.0
Fructose (N)	5	18	<0.002	3.6
LIPIDS				
Lauric acid (12:0) (Y)	2	0.8	<0.002	0.4
Myristic acid (14:0) (Y)	4	1	<0.002	0.3
Palmitic acid (16:0) (Y)	41	12	<0.002	0.3
Linoleic acid (18:2 9*Z*,12*Z*) (Y)	5	2	<0.002	0.4
Oleic acid (18:1 9*Z*) (Y)	27	7	<0.002	0.3
Stearic acid (18:0) (N)	14	6	<0.002	0.4
Cholesterol (Y)	31	16	<0.002	0.5

* Nonparametric Mann–Whitney U test. *p* values adjusted by Bonferroni correction.

**Table 2:** Metabolites showing a difference in ascites and plasma relative concentration.

Metabolite (Reported Previously in Ascites; Y = Yes, N = No)	Plasma (Median)	Ascites (Median)	* p Value	Ascites/ Plasma Ratio
AMINO ACIDS				
Glycine (Y)	31	43	0.004	1.4
Valine (Y)	61	73	0.004	1.2
Proline (N)	85	136	0.002	1.6
Threonine (N)	12	22	0.002	1.8
Methionine (Y)	9	13	0.004	1.4
Lysine (Y)	17	22	0.004	1.3
Glutamine (Y)	34	62	0.02	1.8
Phenylalanine (Y)	27	34	0.03	1.3
Tyrosine (Y)	65	113	0.004	1.7
OTHER INTERMEDIARY METABOLITES				
Pyruvic acid (Y)	328	532	0.004	1.6
Fumaric acid (N)	1	3	0.02	3.0
Sorbitol (N)	0.4	0.6	0.02	1.5
Phosphate (N)	371	49	0.007	0.1
LIPIDS				
Myristic acid (14:0) (Y)	5	1	0.002	0.2
Palmitic acid (16:0) (Y)	59	12	0.002	0.2
Oleic acid (18:1 9*Z*) (Y)	45	6	0.002	0.1
Vaccenic acid (18:1 11*E*) (N)	4	0.7	0.002	0.2
Stearic acid (18:0) (N)	12	5	0.002	0.4
Cholesterol (Y)	97	21	0.002	0.2

* Nonparametric Wilcoxon matched pairs test. *p* values adjusted by Bonferroni correction.

## Data Availability

The data that support the findings of this study are available from the corresponding author upon reasonable request.

## Supplementary Materials

The following supporting information can be downloaded at: https://www.mdpi.com/article/10.3390/molecules27123935/s1, Table S1: Metabolites detected by GC-MS in ascitic fluid and plasma from ascites patients and peritoneal fluid from postoperative surgical patients; Figure S1: PCA Variances and scores plots for Experiment 1; Figure S2: Leave-one-out cross validation of the PLS-DA models for Experiment 1 (A) and Experiment 2 (B); Figure S3: PCA Variances and scores plots for Experiment 2; Figure S4: ROC analysis for A. combined elevated amino acids and combined diminished lipids in ascites fluid for Experiment 1 and B. combined elevated amino acids and combined diminished lipids in ascites fluid for Experiment 2; Figure S5: Heatmap showing all ascites and plasma samples from Experiment 2 and all metabolite features.

## Sample Availability

Samples of the compounds … are available from the authors.
