# Supplementary material for: A Metabolomic Analysis of Cirrhotic Ascites
Source: Molecules. 2022 Jun 20;27(12):3935. doi: 10.3390/molecules27123935 (PMC9228447; doi:10.3390/molecules27123935)
Supplement: Supplementary file 1 [file molecules-27-03935-s001.zip › molecules-1699051-supplementary.pdf]

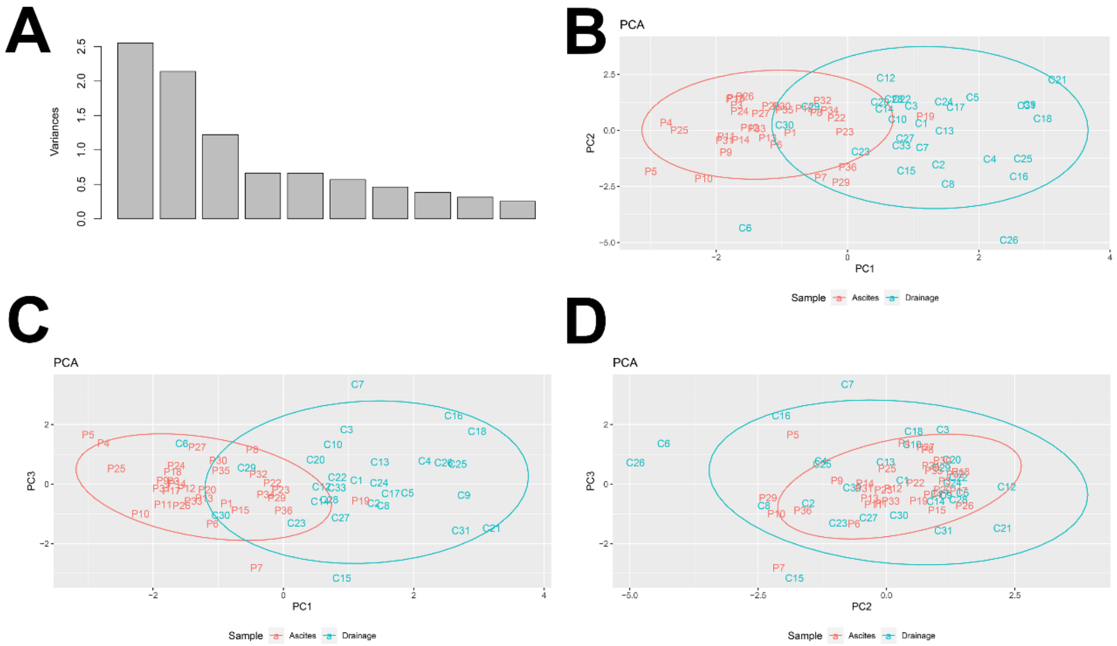


**Figure S1**. PCA Variances and scores plots for Experiment 1

**A**, magnitude of variances; **B**, PCA scores plot principal component 1 (PC1) vs. principal component 2 (PC2) showing 95% confidence ellipses; **C**, PCA scores plot principal component 1 (PC1) vs. principal component 3 (PC3) showing 95% confidence ellipses; **D**, PCA scores plot principal component 2 (PC2) vs. principal component 3 (PC3) showing 95% confidence ellipses. Red ellipse and scores represent cirrhotic ascites patient ascites fluid. Green ellipse and scores represent drainage fluid from control postoperative surgical patients.

A

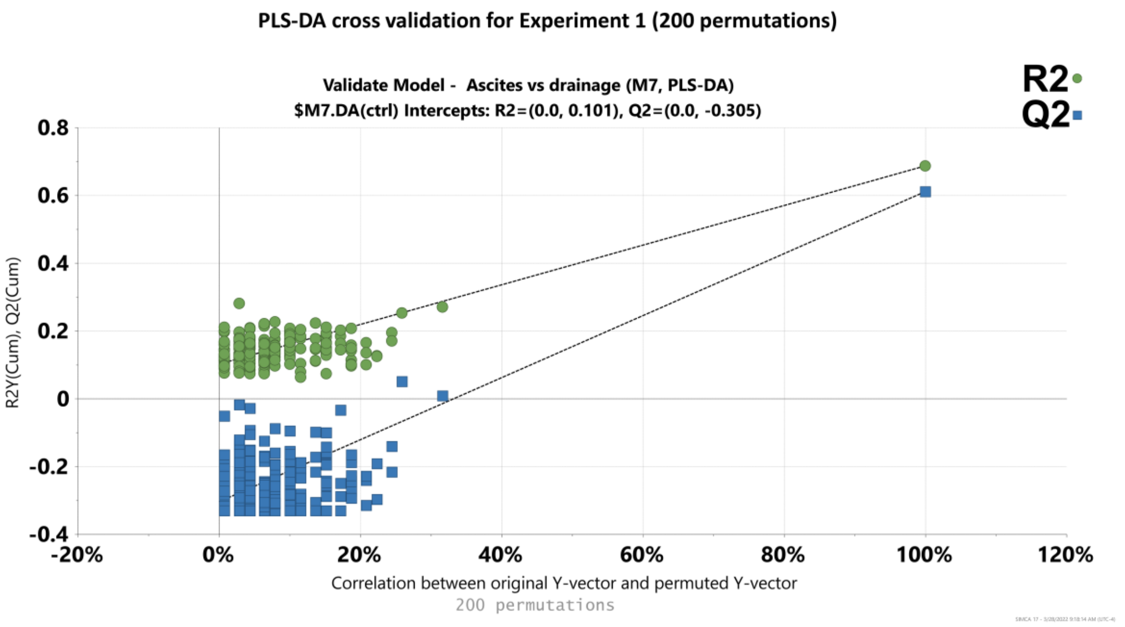


B

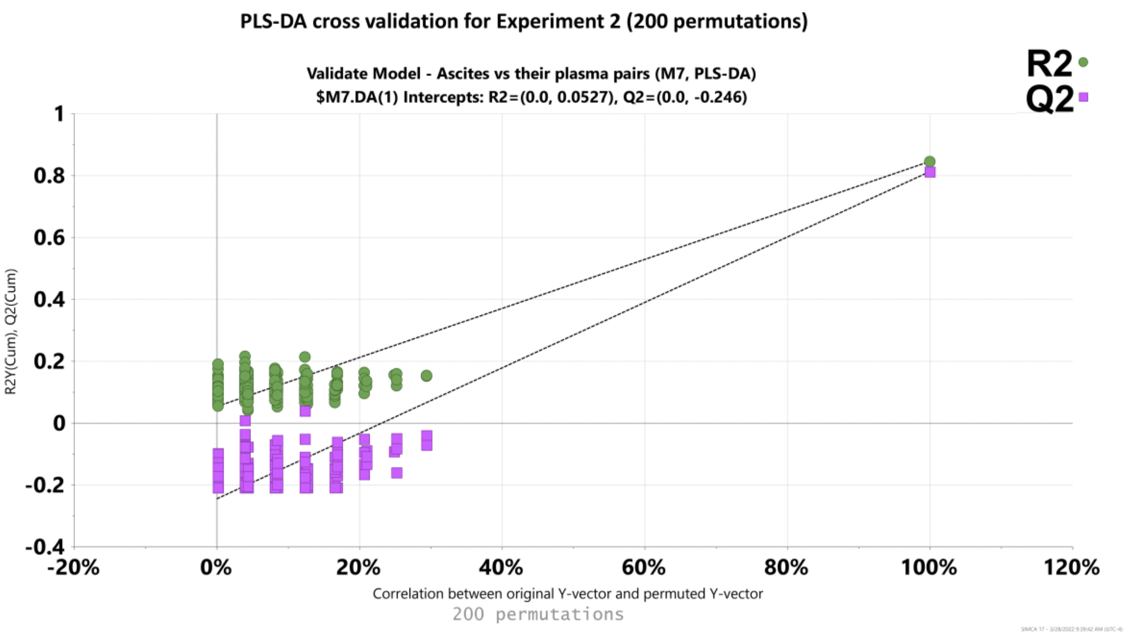


**Figure S2.** Leave-one-out cross validation of the PLS-DA models for Experiment 1 (A) and Experiment 2 (B)

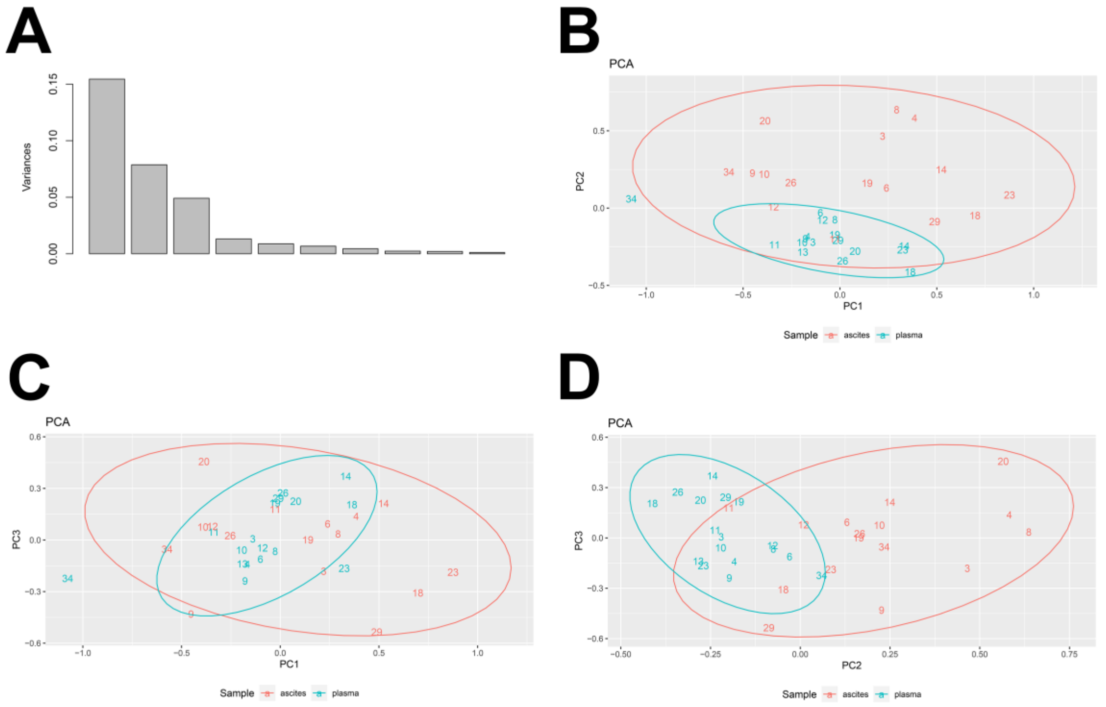


**Figure S3**. PCA Variances and scores plots for Experiment 2

**A**, magnitude of variances; **B**, PCA scores plot principal component 1 (PC1) vs. principal component 2 (PC2) showing 95% confidence ellipses; **C**, PCA scores plot principal component 1 (PC1) vs. principal component 3 (PC3) showing 95% confidence ellipses; **D**, PCA scores plot principal component 2 (PC2) vs. principal component 3 (PC3) showing 95% confidence ellipses. Red ellipse and scores represent cirrhotic ascites patient ascites fluid. Green ellipse and scores represent cirrhotic ascites patient plasmas.

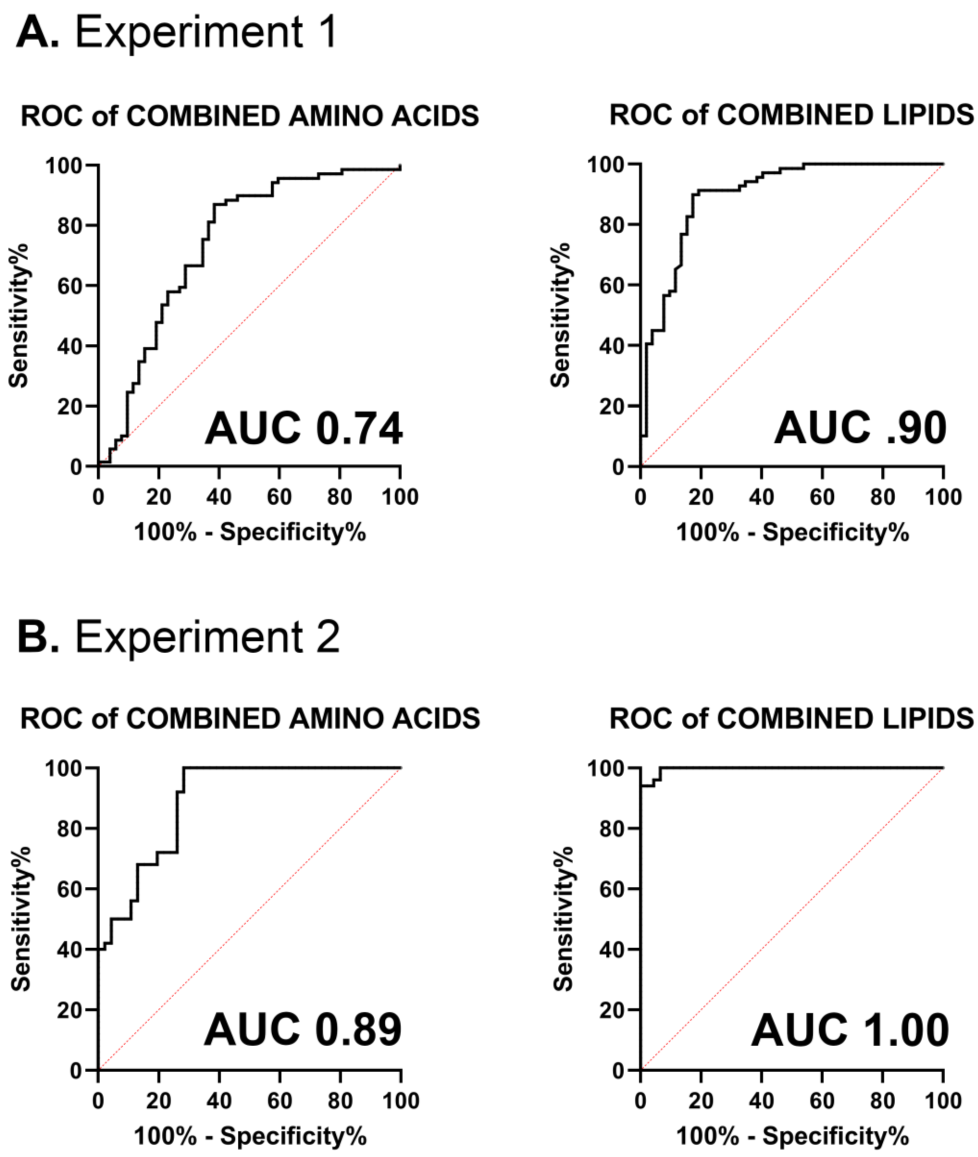


**Figure S4**. ROC analysis for **A.** combined elevated amino acids and combined diminished lipids in ascites fluid for Experiment 1 and **B.** combined elevated amino acids and combined diminished lipids in ascites fluid for Experiment 2

All ROC analyses were significant ($P < 0.0001$).

<u>Experiment 1</u>. Combined elevated amino acids were glutamine, proline, histidine and tyrosine. Combined diminished lipids were lauric, myristic, palmitic, linoleic, oleic and stearic acids, together with cholesterol (Table 1).

<u>Experiment 2</u>. Combined elevated amino acids were glycine, valine, proline, threonine, methionine, lysine, glutamine, phenylalanine and tyrosine. Combined diminished lipids were myristic, palmitic, oleic, vaccenic and stearic acids, together with cholesterol (Table 2).

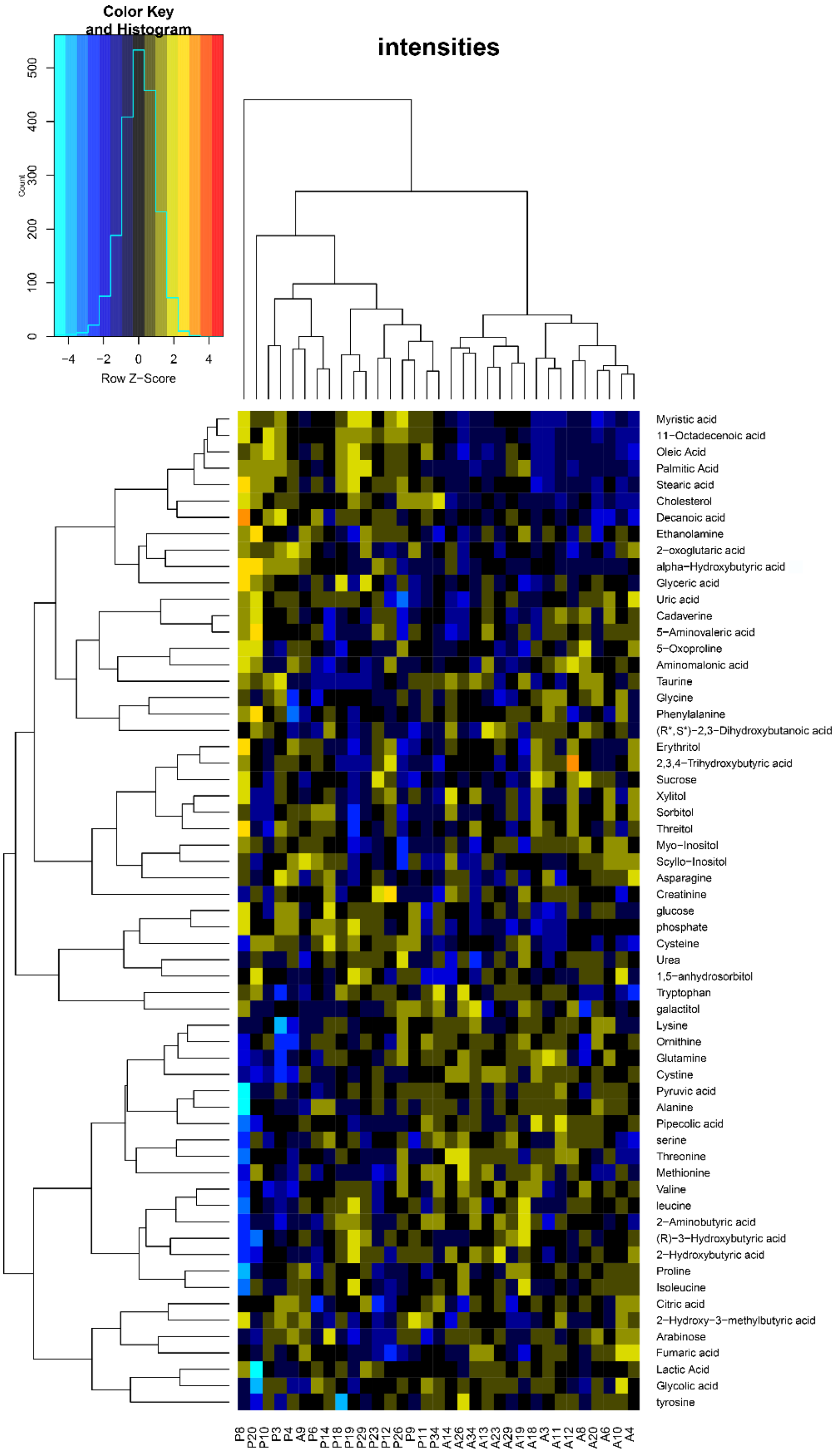


**Figure S5.** Heatmap showing all ascites and plasma samples from Experiment 2 and all metabolite features.

**Table S1.** Metabolites detected by GC-MS in ascitic fluid and plasma from ascites patients and peritoneal fluid from postoperative surgical patients

| Metabolite | Retention time (min) | Derivatives formed |
|---|---|---|
| Ethanolamine | 9.25 | 2TMS |
| Propylene glycol | 9.33 | 2TMS |
| Pyruvic acid | 9.59 | 1TMS |
| Lactic acid | 9.79 | 2TMS |
| Glycolic acid | 9.98 | 2TMS |
| Alanine | 10.43 | 2TMS |
| Glycine | 10.67;13.53 | 2TMS; 3TMS |
| 2-Hydroxybutyric acid | 10.82 | 2TMS |
| 3-Hydroxybutyric acid | 11.30 | 2TMS |
| 2-Hydroxy-3-methylbutyric acid | 11.41 | 2TMS |
| 2-Aminobutyric acid | 11.55 | 2TMS |
| Urea | 11.70;12.51;12.63 | 2TMS; 3TMS |
| 2-Ketoisocaproic acid | 12.12 | 1TMS; 1MOX |
| Valine | 12.19 | 2TMS |
| Leucine | 13.00 | 2TMS |
| Phosphate | 13.10 | 3TMS |
| Isoleucine | 13.32 | 2TMS |
| Proline | 11.59; 13.41 | 1TMS; 2TMS |
| Glyceric acid | 13.83 | 3TMS |
| Fumaric acid | 13.94 | 2TMS |
| 2,3-Dihydroxybutanoic acid | 14.18 | 3TMS |
| Serine | 14.24 | 3TMS |
| Uracil | 14.31 | 2TMS |
| Pipecolic acid | 14.35 | 2TMS |
| Threonine | 14.62 | 3TMS |
| Cadaverine | 15.08 | 3TMS |
| Aminomalonic acid | 15.73 | 3TMS |
| 4-Chlorophenylacetic acid **(ISTD)** | 15.90 | 1TMS |
| Erythritol | 16.21 | 4TMS |
| Methionine | 16.35 | 2TMS |
| 5-Oxoproline | 16.40 | 2TMS |
| 2,3,4-Trihydroxybutyric acid | 16.66 | 4TMS |
| Cysteine | 16.79 | 3TMS |
| Creatinine | 16.88 | 3TMS |
| 2-Oxoglutaric acid | 16.96 | 2TMS; 1MOX |
| 5-Aminovaleric acid | 17.34 | 3TMS |
| Ornithine | 17.45 | 3TMS |
| Glutamic acid | 17.50 | 3TMS |
| Phenylalanine | 17.68 | 2TMS |
| Lauric acid | 17.78 | 1TMS |
| Asparagine | 18.13 | 3TMS |
| Taurine | 18.20 | 3TMS |
| Lysine | 18.50; 20.84 | 3TMS; 4TMS |
| Xylitol | 18.79 | 5TMS |
| Galactitol | 18.84 | 6TMS |
| Glutamine | 19.26 | 3TMS |
| Threitol | 19.40 | 4TMS |
| Citric acid | 19.81 | 4TMS |
| Myristic acid | 19.92 | 1TMS |
| 1,5-Anhydrosorbitol | 20.19 | 4TMS |
| Arabinose | 20.28 | 5TMS; 1MOX |
| Fructose | 20.47; 20.55 | 5TMS; 1MOX<br>5TMS; 1MOX |
| Galactose | 20.62 | 5TMS; 1MOX |

| | | |
|---|---|---|
| Glucose | 20.66; 20.76 | 5TMS; 1MOX<br>5TMS; 1MOX |
| Histidine | 20.88 | 3TMS |
| Tyrosine | 21.03 | 3TMS |
| Mannitol | 21.09 | 5TMS; 1MOX |
| Mannose | 21.44 | 5TMS |
| Gluconic acid | 21.82 | 6TMS |
| Palmitic acid | 21.87 | 1TMS |
| Scyllo-inositol | 22.05 | 6TMS |
| Uric acid | 22.62 | 4TMS |
| Myo-inositol | 22.65 | 6TMS |
| Sorbitol | 22.86 | 6TMS |
| Linoleic acid | 23.42 | 1TMS |
| Oleic acid | 23.46 | 1TMS |
| Vaccenic acid | 23.51 | 1TMS |
| Stearic acid | 23.65 | 1TMS |
| Tryptophan | 23.73 | 3TMS |
| Sucrose | 27.38 | 8TMS |
| Cholesterol | 33.57 | 1TMS |
